# Effect of blanching and ultrasound pretreatment on moisture migration, uniformity, and quality attributes of dried cantaloupe

**DOI:** 10.1002/fsn3.3396

**Published:** 2023-05-02

**Authors:** Tiejian Yuan, Xiaoyan Zhao, Chao Zhang, Peng Xu, Xiaoqiong Li, Zhentao Zhang, Junling Yang, Yaoyang Liu, Yan He

**Affiliations:** ^1^ College of Electromechanical Engineering Qingdao University of Science and Technology Qingdao China; ^2^ Technical Institute of Physics and Chemistry Chinese Academy of Sciences Beijing China; ^3^ Key Laboratory of Vegetable Postharvest Processing Ministry of Agriculture and rural affairs Beijing China; ^4^ Xi'an Jiaotong University Xi'an China; ^5^ Jiangxi CAS Pharmaceutical Engineering Technology Co., Ltd. Nanchang China

**Keywords:** browning, crusting, moisture migration, pretreatment, uniformity

## Abstract

To overcome problems of browning and crusting during the pretreatment process and provide theoretical guidance for cantaloupe convection drying at 80°C, the effects of blanching (BL) and ultrasonic (US) treatments were examined. The effects of various BL (5, 10, and 15 s) and US (10, 20, 30, and 40 min) durations on convection drying were tested. The moisture ratio, drying rate, moisture effective diffusivity, color, browning, nuclear magnetic resonance characteristics, and texture were assessed. Compared with the control group, the maximal decreases in the drying time of BL and US pretreatment groups were 40% and 33.3%, respectively. BL and US pretreatments significantly increased the effective diffusion coefficient and shortened the drying time because of the destruction of the cell structure. Low‐field nuclear magnetic resonance analysis showed that free water is mainly lost during the initial drying stage, while solidified water is mainly lost during middle and late stages. According to the results of magnetic resonance imaging, the moisture distribution shows that cavitation from US acts on internal tissue, while BL disrupts the structure of external tissue. Texture data define the area enclosed by S_C‐D_ as uniform. After BL and US pretreatment, the hardness of dried cantaloupe decreased and the uniformity increased significantly. The best pretreatment process for cantaloupe at 80°C was 10 min of US. These findings provide a reference for testing in the industrial production of dried cantaloupe and are deeply relevant for practice.

## Graphical abstract

Detailed moisture change during drying is revealed. Characterization of material drying uniformity by S_C‐D_ area. The optimal pretreatment group is US 10 min.

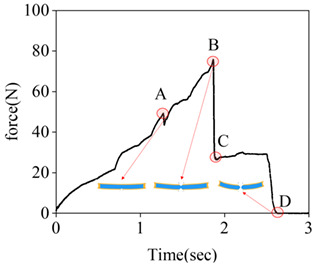

## 1 INTRODUCTION

According to its botanical classification, the cantaloupe is a cucurbitaceous melon (Shang et al., 2020) and the fifth most important fruit worldwide in terms of production (31,166,896 tons in 2021). It is widely cultivated in Xinjiang, China, and frequently consumed because it is rich in vitamins and carbohydrates, featuring the characteristics of sweetness, juiciness, and nutritiousness (Benmeziane et al., 2018). However, fresh cantaloupe has high moisture contents of up to 85%–90%, which greatly increases its susceptibility to microbial infection. Cantaloupe spoils and rots within 10–15 days if not processed in time, leading to a large number of discarded cantaloupes, which negatively impacts both the cantaloupe industry and the environment (Yao et al., 2020).

Convection drying—the main technology for preserving perishable fruits—can quickly reduce the moisture content of cantaloupe and effectively solve the above‐mentioned problems (Naknean et al., 2012). During convective drying, heat is mainly transferred to the surface of low‐temperature materials through high‐temperature airflow, and the temperature gradient causes heat exchange. During the heating of the material, the proportion of water vapor will change because of phase transition, and the emerging concentration gradient will cause the water to diffuse to the outside. Previous studies have shown that when temperatures are increased from 50 to 80°C, the convection drying time of cantaloupe can be greatly shortened. However, at a temperature of 80°C, two problems will emerge: First, at this temperature, cantaloupe will be subject to significant browning. In the processing of fruits and vegetables, browning is undesirable as it not only affects the flavor but also reduces the nutritional value. Second, during the later stage of drying, because of differences in water diffusion rates, the material surface will shrink and a crust will appear. This phenomenon is not conducive to the external diffusion of internal moisture, resulting in poor uniformity and higher energy consumption. It is very important to take corresponding measures to optimize the convection drying process of cantaloupe at 80°C. To optimize the drying process, one of the ways in which moisture diffusion rates can be increased can be achieved through the use of pretreatments such as application of blanching (BL) and ultrasound (US) treatments.

Blanching and US are the main pretreatment methods employed before fruits and vegetables are dried (Zhu et al., 2021). BL is a heat treatment method in which a material is either placed in hot water or exposed to steam for a certain period of time. BL can shorten the drying time and improve the state of materials, which has been shown to positively impact the browning behavior of persimmons (Oshima et al., 2021), seaweeds (Susanto et al., 2017), and carrots (Imaizumi et al., 2017). US is a new and promising non‐thermal processing technology in food processing. It can affect the drying characteristics of the materials and change dynamic characteristics based on the mass and heat transfer process (Schoessler et al., 2012). US can induce a series of effects, such as the sponge effect, cavitation, and micro channels, which greatly affect mass transfer and cell wall permeability (Ren et al., 2018). Because of the non‐thermal characteristics of US, compared with the drying results of untreated materials, the adoption of US can shorten the drying time while retaining nutrients, which has a certain effect on browning and color difference of heat‐sensitive materials (Monica et al., 2018). However, these two kinds of pretreatment measures are relatively rarely used for convection drying of cantaloupe. In pursuit of shorter drying time, a method needs to be found that can inhibit both browning and crusting. In addition, conventional texture analysis (using hardness, brittleness, and toughness) failed to characterize the overall structure distribution of the finished product and the meaning of changes in the curve in the testing process. At present, there is no quality analysis method that can directly reflect the crusting phenomenon and uniformity. Therefore, it is necessary to deeply analyze relevant changes and causes of the texture curve on the basis of these data. Such an analysis provides a reference point for product drying in industrial production and can be more clearly integrated with production and applied in practice.

The purpose of this study was to test the capability of two pretreatment methods to inhibit browning and crusting of cantaloupe during convection drying at 80°C, and to identify a new method to evaluate the uniformity of dried cantaloupe slices. Through the analysis of drying kinetics, color difference, browning, nuclear magnetic resonance characteristics, and texture, the best processing technology and mechanism for improving the uniformity of cantaloupe were identified.

## 2 MATERIALS AND METHODS

### 2.1 Materials

Fresh cantaloupes were purchased from a local market in Beijing, China. To ensure the uniformity of experimental materials, cantaloupes with equatorial diameter of 10 ± 2 cm, a weight of 2.5 ± 0.5 kg, and no mechanical damage were artificially selected. To avoid quality deterioration, all cantaloupes were stored in a refrigerator at a relative humidity of 95% and a temperature of 4 ± 1°C for no longer than 3 days. Before the experiment, the stored cantaloupes were taken out of the refrigerator and cut into slices with a thickness of 5.0 ± 1.0 mm and a cross‐sectional area of 40.0 × 40.0 mm. The initial moisture content of fresh cantaloupes was estimated by AOAC (1990, no. 950.46), and its wet basis was 88.00%. Cantaloupe slices with uniform length and size were subjected to different pretreatments.

### 2.2 Method and conditions

#### 2.2.1 Blanching pretreatment

About 200 g of cantaloupe slices were immersed in a hot water bath at 100°C for either 5, 10, or 15 s. In this study, it was found that a longer BL time would cause serious tissue damage. Then, the moisture on the surface of cantaloupe slices was absorbed by gauze. Afterward, the drying process was initiated. The ratio of sample to water was 1:15 w/w to reduce the temperature change during BL. In this study, cantaloupe slices without any pretreatment were used as control.

#### 2.2.2 Ultrasound pretreatment

About 200 g of cantaloupe slices were immersed in an US water bath with a size of 500 × 300 × 150 mm (KQ‐500DE, Kunshan Shumei) at 40 kHz and 500 W/20°C for 10, 20, 30, or 40 min. Then, the moisture on the surface of cantaloupe slices was absorbed by gauze. Afterwards, the drying process was initiated. As in BL treatment, cantaloupe slices without any pretreatment were used as control.

### 2.3 Hot‐air drying

Cantaloupe samples were dried with a heat pump dryer developed by the Technical Institute of Physics and Chemistry of the Chinese Academy of Sciences. The air temperature, speed, and relative humidity were fixed at 80°C, 1.0 m/s, and 10%, respectively. An electronic balance (PB8001‐L, Mettler Toledo, Switzerland) was installed in the dryer to measure the water loss of samples during drying. The same number of BL pretreated, US pretreated, and untreated cantaloupe slices were placed on three identical stainless‐steel trays during the experiment. The weight of samples was monitored with the electronic balance at set time intervals until the wet basis of samples reached 10%, at which point, drying was stopped.

### 2.4 Moisture content and drying rate

The moisture content (MR) and drying rate of cantaloupe slices in the thin‐layer drying process were calculated based on the experimental quality data. The following formulae are used:

(1) $$\mathrm{MR}=\frac{M-M_{e}}{M_{0}-M_{e}}$$

(2) $$\text{Drying rate}=\frac{M_{t+\mathrm{dt}}-M_{t}}{\mathrm{dt}}$$

where M is the moisture content in the drying process; $M_{0}$ is the initial moisture content; $M_{e}$ is the equilibrium moisture content; $M_{t}$ is the moisture content at time *t*; $\;M_{t+\mathrm{dt}}$ is the moisture content at time *t + dt*; all units of the moisture contents in different states are kg water/kg dry matter; and dt is the drying time (min).

### 2.5 Effective moisture diffusivity

The diffusion of moisture in materials during drying is a complex process consisting of surface diffusion, capillary flow, and molecular diffusion. The strength of this process can be expressed by the effective moisture diffusivity (*D*_*eff*_). The first term of Fick's diffusion equation was used to calculate the effective moisture diffusivity for a slice, as shown in Equation (3):

(3) $$\mathrm{MR}=\frac{8}{\pi^{2}}\exp\left(-\pi^{2}\frac{D_{\mathrm{eff}}t}{4L^{2}}\right)$$

where *D*_*eff*_ is the effective moisture diffusion (m^2^/s) and *L* is half the thickness of the dried cantaloupe slice (m).

### 2.6 Color measurement

A spectrophotometer (LabScan XE spectrophotometer, Hunter Associates Laboratory, Inc.) was used to measure the color of each group of cantaloupe slices during the drying process at hourly intervals. Untreated cantaloupe slices were used as controls.

The CIE L*a*b* system was adopted for color measurement, and poor brightness (*ΔL*) was calculated by Equation (4):

(4) $$\Delta L=L^{*}-{L_{0}}^{*}$$

The chromaticity difference was calculated by Equations (5) and (6):

(5) $$\Delta a=a^{*}-{a_{0}}^{*}$$

(6) $$\Delta b=b^{*}-{b_{0}}^{*}$$

The total color difference (*ΔE*) was calculated by Equation (7):

(7) $$\Delta E=\sqrt{\Delta L^{2}+\Delta a^{2}+\Delta b^{2}}$$

In the experiment, L*, a*, and b* are the color parameters of control, BL, or US treated and dried cantaloupe, and *L*_*0*_ *, *a*_*0*_ *, and *b*_*0*_ * are the color parameters of fresh cantaloupes.

Nine slices of each sample were tested, and the arithmetic average was used for data analysis.

### 2.7 Determination of browning degree

The browning degree of cantaloupe was evaluated using the spectrophotometric method. Reagents were prepared according to the following procedure: For 0.1 M phosphate buffer (pH = 6.8), 71.64 g of disodium hydrogen phosphate was placed into a beaker, and a small amount of water was added. The mixture was heated and stirred until disodium hydrogen phosphate had completely dissolved, keeping a constant volume of 1000 mL. Further, 31.2 g of sodium dihydrogen phosphate was placed into the beaker, a small amount of water was added, and the volume was diluted to 1000 mL. Moreover, 49 mL of disodium hydrogen phosphate solution and 51 mL of sodium dihydrogen phosphate solution were diluted to 200 mL.

The measurement method used the following procedure: A total of 10 g of dried cantaloupe samples were ground into a powder, 50 mL of phosphate buffer (W: V = 1:1) was added, and the mixture was precooled at 4°C for 30 min. Then, the pulp was quickly beaten, filtered by suction, and the temperature was set to 4°C. After, the mixture was centralized at a central force of 7104 *g* for 15 min, and the supernatant was taken to measure the absorbance at a wavelength of 410 nm. The measurement was repeated three times and took the arithmetic average.

### 2.8 NMR and MRI analyses

A low‐field nuclear magnetic resonance (LF‐NMR) analyzer (NMI20‐025 V‐I, Newman Company) was used to measure the transverse relaxation time (*T*_*2*_) of different treatment groups according to different drying times. Dried cantaloupe samples were cut in half at room temperature (25°C) and then transferred to a nuclear magnetic resonance (NMR) glass tube with a diameter of 25 mm. The transverse relaxation time (*T*_*2*_) was measured by the Carr–Purcell–Meiboom–Gill pulse sequence. The relevant parameters were as follows: echo time (TE), 1 ms; waiting time (TW), 4000 ms; scan number (NS), 8; and echo number (NECH) 10,000.

Magnetic resonance imaging (MRI) was used to examine the distribution of water in the sample. The relevant parameters of all images were as follows: slice width 20 mm; slice gap,1.0 mm, read size, 256; phase size, 192; repetition time (TR), 500 ms; and echo time (TE), 20 ms.

### 2.9 Texture measurement

A texture analyzer (TA‐XT plus, Stable Micro System) was used to measure the texture properties of cantaloupe slices. The force on the probe and the time of probe movement during compression were recorded. The hardness and uniformity of differently treated samples were analyzed, where hardness is the maximum force during compression, and uniformity is the work done by the force after the highest peak fracture. For texture measurement, dry and finished products with relatively uniform shapes and sizes from different treatment groups were selected and placed under the texture analyzer HDP/BSK probe for texture testing. The texture analyzer parameters were set as follows: pretest rate, 2 mm/s; test rate, 1 mm/s; test completion rate was identical to the test rate; the pressing distance was 5 mm; the data collection rate was 400 PPS; each set of sample data was collected 10 and took the arithmetic average.

### 2.10 Statistical analysis

All analyses were conducted in triplicate. The data were analyzed using SPSS statistics software (version 21.0, SPSS Inc.). Statistical analysis was performed with Origin (version 2022; Origin‐Lab).

## 3 RESULTS AND DISCUSSION

### 3.1 Drying kinetics

**FIGURE 1:**
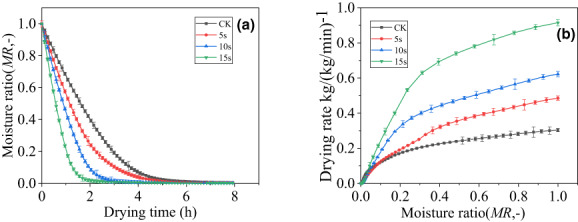
(a) Moisture ratio of cantaloupe pretreated by blanching (BL) at different times during hot‐air drying at 80°C. (b) Relationship between drying rate and moisture ratio of cantaloupe pretreated by BL at different times during hot‐air drying at 80°C. ‐■‐AD 80°C; ‐●‐BL 5 s + AD 80°C; ‐▲‐ BL 10 s + AD 80°C; and ‐▼‐ BL 15 s + AD 80°C.

**FIGURE 2:**
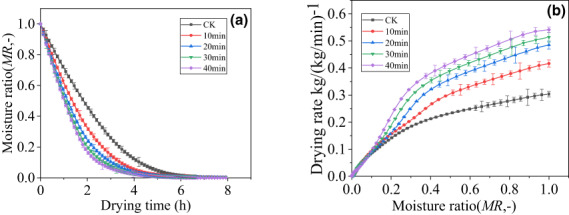
(a) Moisture ratio of cantaloupe pretreated by ultrasonic (US) treatment at different times during hot‐air drying at 80°C. (b) Relationship between drying rate and moisture ratio of US pretreated cantaloupe for different times during hot‐air drying at 80°C. ‐■‐AD 80°C; ‐●‐US 10 min + AD 80°C.

As shown in Figures 1a and 2a, *MR* drops rapidly in the early stage of drying and slows down in the later stage of drying. Both the BL pretreatment group and the US pretreatment group showed effectively shortened drying time. Compared with the drying time required by the control (CK), the drying time required to reach equilibrium moisture content (<0.05 kg water/kg dry basis) for the three BL pretreatment groups (5, 10, and 15 s) and the four US pretreatment groups (10, 20, 30, and 40 min) decreased by 6.7%, 24.7%, 40%, 12%, 21%, 26.7%, and 33.3%, respectively.

As shown in Figures 1b and 2b, the drying rate of each group decreased with decreasing MR. Compared with CK, the drying rate of the three BL groups (5, 10, and 15 s) and the four US pretreated groups (10, 20, 30, and 40 min) increased by 59.8%, 105.1%, 201.4%, 37.2%, 56.9%, 69.3%, and 78.4%, respectively.

The drying rate of the product depends not only on the drying conditions but also on other processes before and after drying. For cantaloupe, encrustation occurs during traditional hot‐air drying. The main reason is that the high sugar content of cantaloupe will cover the surface and hinder water migration during the drying process (Xu, Peng, Yang, et al., 2021). BL pretreatment and US pretreatment increase the drying and dehydration speed by changing the physical properties of cantaloupe tissue, thereby improving the quality of the finished product. BL damages and ruptures cell walls and cell membranes, which increases the speed of water movement and thus, the drying rate of cantaloupe (Ando et al., 2016). Similar phenomena were also observed in the hot‐air‐drying processes of cut persimmons (Oshima et al., 2021), apricots (Deng et al., 2018), and wolfberries (Zhao et al., 2019). The US pretreatment method where fresh cantaloupe slices are immersed in liquid with high‐power US action will cause cavitation effects. Cavitation produces a large number of small bubbles in the liquid. These small bubbles not only grow until they suddenly burst and form shock waves, but also cause a series of changes in the internal tissue of the cantaloupe, making it easier to remove water. US pretreatment can effectively enhance the mass transfer process and increase the drying rate. This feature has been reported in the dehydration and drying of apples (Aleksandra et al., 2015), kiwi slices (Prithani & Dash, 2020), and Chinese yams (Li et al., 2020). As motioned above, both BL and US pretreatment methods can effectively increase the drying rate of cantaloupe, shorten the drying time, and solve the problem of crusts to a certain extent.

### 3.2 Effective moisture diffusivity

As shown in Table 1, the effective moisture diffusivity of CK was 2.86 × 10^−10^, that of the BL treatment group was 2.32 × 10^−9^–2.93 × 10^−9^, and that of the US treatment group was 9.96 × 10^−10^–2.22 × 10^−9^. Both BL and US treatments significantly increased the effective moisture diffusivity of dried cantaloupe at 80°C (Óscar et al., 2017), and the effective moisture diffusivity increased with extended treatment time.

**TABLE 1:** Effective moisture diffusivity and *R* ^2^ of different treatment groups.

Sample	*D*_eff_ (m^2^/s)	*R* ^2^
CK	2.86 × 10^−10^	.9365
BL (5 s)	2.32 × 10^−9^	.9824
BL (10 s)	2.62 × 10^−9^	.9956
BL (15 s)	2.93 × 10^−9^	.9991
US (10 min)	9.96 × 10^−10^	.9887
US (20 min)	1.13 × 10^−9^	.9876
US (30 min)	1.98 × 10^−9^	.9691
US (40 min)	2.22 × 10^−9^	.9868

The reason may be that a longer pretreatment time causes greater change in the material, which in turn, allows more moisture to pass through the dried product per unit of time during convection drying. As a hygroscopic porous medium, the diffusion of water in a solid phase generally occurs in the deceleration period and mainly happens in the forms of capillary flow and surface diffusion (Mbegbu et al., 2021). US cavitation and BL broaden the capillary channels through which part of the water is transferred inside the porous medium, which increases the diffusion rate. The resistance of surface diffusion mainly originates from the binding of cells to bound water. Pretreatment can damage and break the cell walls and membranes of plant cells to a certain extent, making it easier for internally bound water to diffuse and escape from the surface.

### 3.3 Color

Color is one of the most important evaluation indicators of dry products (Xu, Peng, Yuan, et al., 2021), which not only determines the quality of products but also affects the acceptability of products by consumers.

As shown in Table 2, compared with the data after drying the CK, the total color difference *ΔE* of each treatment group increased except for the BL 15 s treatment group. Usually, compared with fresh cantaloupe, the smaller the *ΔE*, the closer the sample is to the original color, and the stronger the acceptability of consumers. However, drying will change L*, a*, and b* to varying degrees, which will affect the total color difference *ΔE*. Research has shown that the higher L*, the higher the brightness of the product, and more consumers find it attractive; the value of a* is related to the oxidation of polyphenols and the Maillard reaction in the hot‐air‐drying process (Baeghblali et al., 2019). The decrease in a* represents the decrease in enzymatic browning. In each treatment group, L* and b* increased, while a* decreased (Geng et al., 2022).

**TABLE 2:** Color measurement of fresh and hot‐air dried samples in each treatment group.

Index	L*	a*	b*	△L*	△a*	△b*	△*E*
Fresh	61.11 ± 0.01^h^	8.79 ± 0.32^i^	22.30 ± 0.68^i^	–	–	–	–
CK	61.51 ± 0.34^g^	16.54 ± 0.49^b^	42.9 ± 0.51^g^	0.4^d^	7.74^b^	20.59^e^	22.01^g^
BL							
5 s	68.29 ± 1.2^c^	15.59 ± 0.98 ^f^	46.94 ± 0.63^d^	7.18^b^	6.79^d^	24.63^c^	26.54^d^
10 s	70.95 ± 0.79^a^	12.87 ± 0.28^h^	49.18 ± 0.96^b^	9.83^a^	4.07^f^	26.88^b^	28.91^b^
15 s	50.68 ± 1.06^i^	19.27 ± 1.86 ^a^	35.51 ± 0.63^h^	−10.4^e^	10.47^a^	13.21^f^	19.82^h^
US							
10 min	70.42 ± 0.96^b^	15.34 ± 0.63^g^	48.69 ± 0.47^c^	9.31^a^	6.54^e^	26.38^b^	28.73^c^
20 min	63.74 ± 0.53^d^	16.06 ± 0.85^d^	51.11 ± 0.83^a^	2.63^c^	7.26^bc^	28.81^a^	29.82^a^
30 min	61.97 ± 0.49^e^	15.89 ± 1.31^e^	45.09 ± 0.37^f^	0.85^d^	7.09^c^	22.79^d^	23.88^f^
40 min	61.63 ± 0.44^f^	16.32 ± 0.44^c^	46.60 ± 0.69^e^	0.52^d^	7.52^b^	24.3^c^	25.44^e^

*Note*: Different values are shown as significantly different (*p* < .05).

In addition, the lightness difference (ΔL*) in Table 4 shows that the lightness (L*) after drying can be improved by BL and US pretreatments for a certain time. It is worth noting that long‐term BL will reduce the brightness of dried products. Especially when the BL was 15 s, the lightness of the finished products will be significantly reduced. This phenomenon was also reported in related research on yams (Xiao et al., 2017). As BL can cause deactivation of polyphenol oxidase, it can protect the color to a certain extent. However, temperature is the main factor of the Maillard reaction, and BL for a long time will lead to the occurrence of this reaction and the deterioration of color. Notably, US exposure does not trigger the Maillard reaction. Therefore, the main factor preventing unnecessary color change is the inactivation of polyphenol oxidase caused by US (Geng et al., 2023).

Overall, compared with CK, a certain period of BL and US pretreatments can effectively improve the color appearance of the finished product. Based on an analysis of experimental data, the recommended pretreatment processes are BL 10 s and US 10 min.

### 3.4 Browning

**FIGURE 3:**
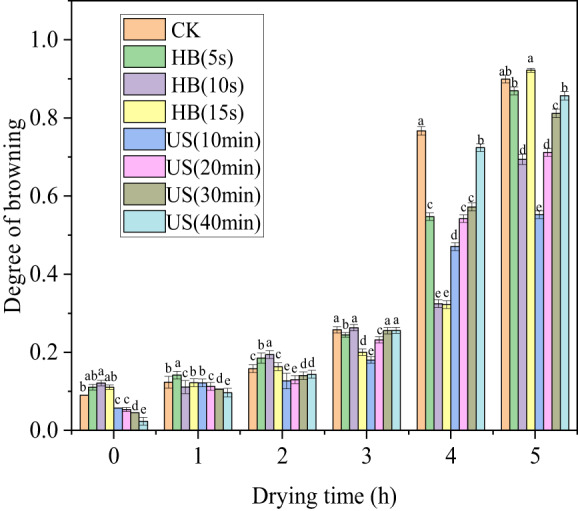
Histogram of changes in browning degree during the drying process for each treatment group.

Browning is a limiting factor affecting sensory quality and value. As shown in Figure 3, the browning of cantaloupe gradually deepened with the drying progress. Compared with CK, browning of other treatment groups decreased except for the BL 15 s treatment group, indicating that BL and US treatments for a certain time can inhibit browning during the drying process. With extended BL treatment time, browning first decreased and then increased, indicating that the inhibitory effect of BL on browning first increased and then decreased. BL for 10 s achieved the best browning inhibition effect and the browning degree was reduced by 22.83%. With the extension of US treatment time, the degree of browning continued to increase, and the inhibitory effect on browning gradually decreased. In all treatment groups, the browning inhibition effect of US for 10 min was the best and the browning degree could be reduced by 38.58%.

Polyphenol oxidase activity causes enzymatic browning reactions in fruits. The results of this study suggest that US pretreatment could inhibit browning of cantaloupe by reducing the activity of polyphenol oxidase. Browning decreased with lengthening US times from 10 to 40 min. When the treatment time exceeds this range, the effect does not change significantly. To avoid unnecessary waste, US pretreatment for 40 min is selected as the optimal treatment to inhibit browning.

### 3.5 Relaxation characteristics determined by LF‐NMR

To examine the effects of BL and US pretreatments on the internal moisture content of cantaloupe slices during the drying process, their T_2_ values were monitored by LF‐NMR.

**FIGURE 4:**
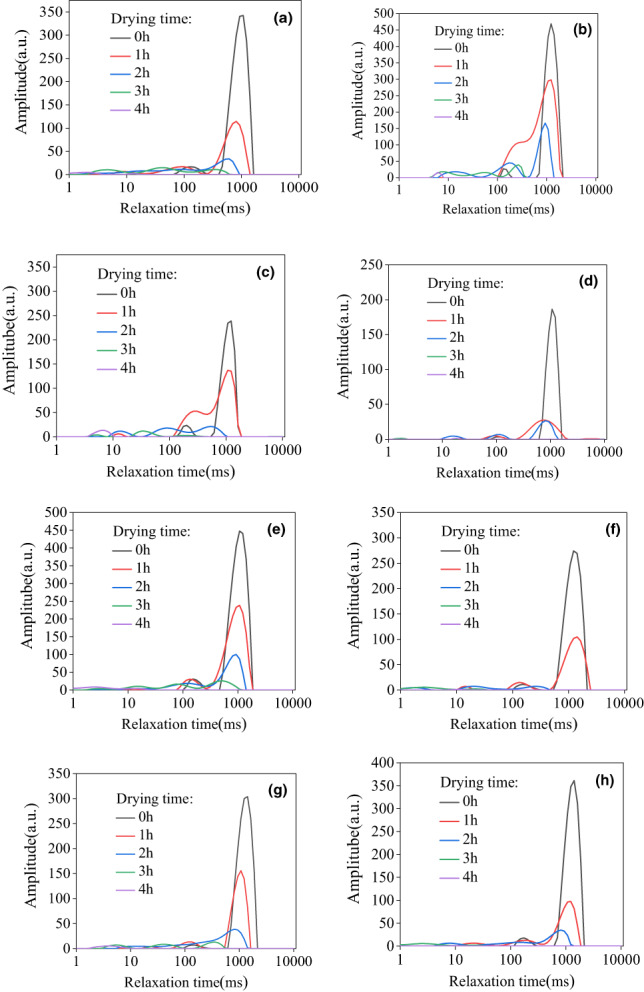
T_2_ relaxation spectrum of cantaloupe in different treatment groups during the drying process. (a) CK; (b, c, and d) 5, 10, and 15 s BL treatment groups, respectively; and (e, f, g, and h) 10, 20, 30, and 40 min US treatment groups, respectively.

As shown in Figure 4a–d, compared with CK (a), the signal amplitude of the 5 s BL pretreatment group (b) increased significantly, while that of the 10 s (c) and 15 s (d) BL pretreatment groups decreased significantly. The signal amplitude increased with decreasing time. The reason for this result may be that a period of high temperature will promote water activities and increase the proportion of free water. However, with increasing BL time, the duration of the heat transfer process at 100°C also increases. In response, some cells on the surface will break, resulting in a large amount of free water outflow, which greatly reduces the content of free water.

As shown in Figure 4a,e,f,g,h, compared with CK (a), the signal amplitude of the US 10 min treatment group (e) increased significantly. In other US treatment groups, the signal amplitude of initial drying increased gradually with extended US time. The drying process after US treatment was faster, and the proportions of free water migration and free water loss were larger. The drying rate was accelerated, which is caused by the cavitation effect of US. During the US pretreatment process, the water inside the cantaloupe will produce tensile stress and form negative pressure, thus changing the cavitation substructure inside the cantaloupe. Because of the influence of pores, the outward diffusion rate of water and the drying rate of cantaloupe were accelerated.

Table 3 shows the initial and final proton transverse relaxation times (T_2_) and amplitudes of different groups. Among them, T21 (0.01–10 ms), T22 (10–100 ms), and T23 (100–1000 ms) were related to bound water, immobilized water, and free water in the cell wall, respectively, which were defined as the water contents of the cytoplasm, extracellular space, and vacuole, respectively (Sya et al., 2020).

**TABLE 3:** Initial and final proton transverse relaxation time (T_2_) and amplitude of different groups.

	T21 (ms)	A21 (%)	T22 (ms)	A22 (%)	T23 (ms)	A23 (%)
Initial						
CK	–	–	92.19 ± 3.94	6.85 ± 0.76^a^	533.67 ± 72.33	93.15 ± 0.63^f^
BL (5 s)	–	–	84.33 ± 2.99	5.44 ± 0.31^b^	533.67 ± 72.31	94.56 ± 0.71^e^
BL (10 s)	–	–	80.65 ± 2.34	2.79 ± 0.09^ef^	613.59 ± 86.67	97.21 ± 0.54^bc^
BL (15 s)	–	–	65.79 ± 1.75	1.58 ± 0.15^g^	533.67 ± 72.36	98.42 ± 0.62^a^
US (10 min)	–	–	79.65 ± 2.16	4.66 ± 0.1^bc^	464.15 ± 68.74	95.31 ± 0.34^de^
US (20 min)	–	–	83.71 ± 2.41	3.78 ± 0.67^cd^	613.59 ± 86.51	96.21 ± 0.87^cd^
US (30 min)	–	–	86.97 ± 2.56	3.29 ± 0.3^de^	533.67 ± 71.61	96.70 ± 0.24^c^
US (40 min)	–	–	86.97 ± 2.55	1.95 ± 0.77^fg^	613.59 ± 86.3	98.04 ± 0.34^ab^
End						
CK	0.05 ± 0.66	66.67 ± 0.91^e^	19.32 ± 1.77	33.32 ± 0.49^a^	–	–
BL (5 s)	0.05 ± 0.34	79.06 ± 0.68^d^	14.17 ± 1.72	20.93 ± 0.44^b^	–	–
BL (10 s)	4.64 ± 1.68	100^a^	–	–	–	–
BL (15 s)	3.05 ± 1.09	100^a^	–	–	–	–
US (10 min)	0.14 ± 0.16	88.31 ± 0.32^c^	12.32 ± 1.63	11.68 ± 0.13^c^	–	–
US (20 min)	1.52 ± 0.2	98.61 ± 0.44^b^	14.17 ± 1.55	1.38 ± 0.8	–	–
US (30 min)	0.12 ± 0.68	100^a^	–	–	–	–
US (40 min)	0.05 ± 0.24	100^a^	–	–	–	–

*Note*: Different values are shown as significantly different (*p* < .05).

Significance analysis was performed on the peak amplitude of the initial and final water contents of each group. The data presented a significant difference (*p* < .05), implying that the BL and US pretreatments had a significant impact.

Compared with CK, both BL and US pretreatments increased the proportion of free water, which further increased with the extension of treatment time. The data of each treatment group at the end of drying were analyzed. Compared with CK, BL and US pretreatments effectively reduced the proportion of immobilized water, which decreased with the extension of the treatment time (Xu et al., 2017).

The water removed during the early stage of the drying process was mainly free water in the vacuole. With the passage of drying time, immobilized water was gradually removed from the extracellular space, thus increasing the proportion of bound water. The application of BL and US pretreatments improved the effective diffusion coefficient of water, promoted the diffusion of water, and achieved a more complete drying.

### 3.6 MRI analysis of drying cantaloupe slices

**FIGURE 5:**
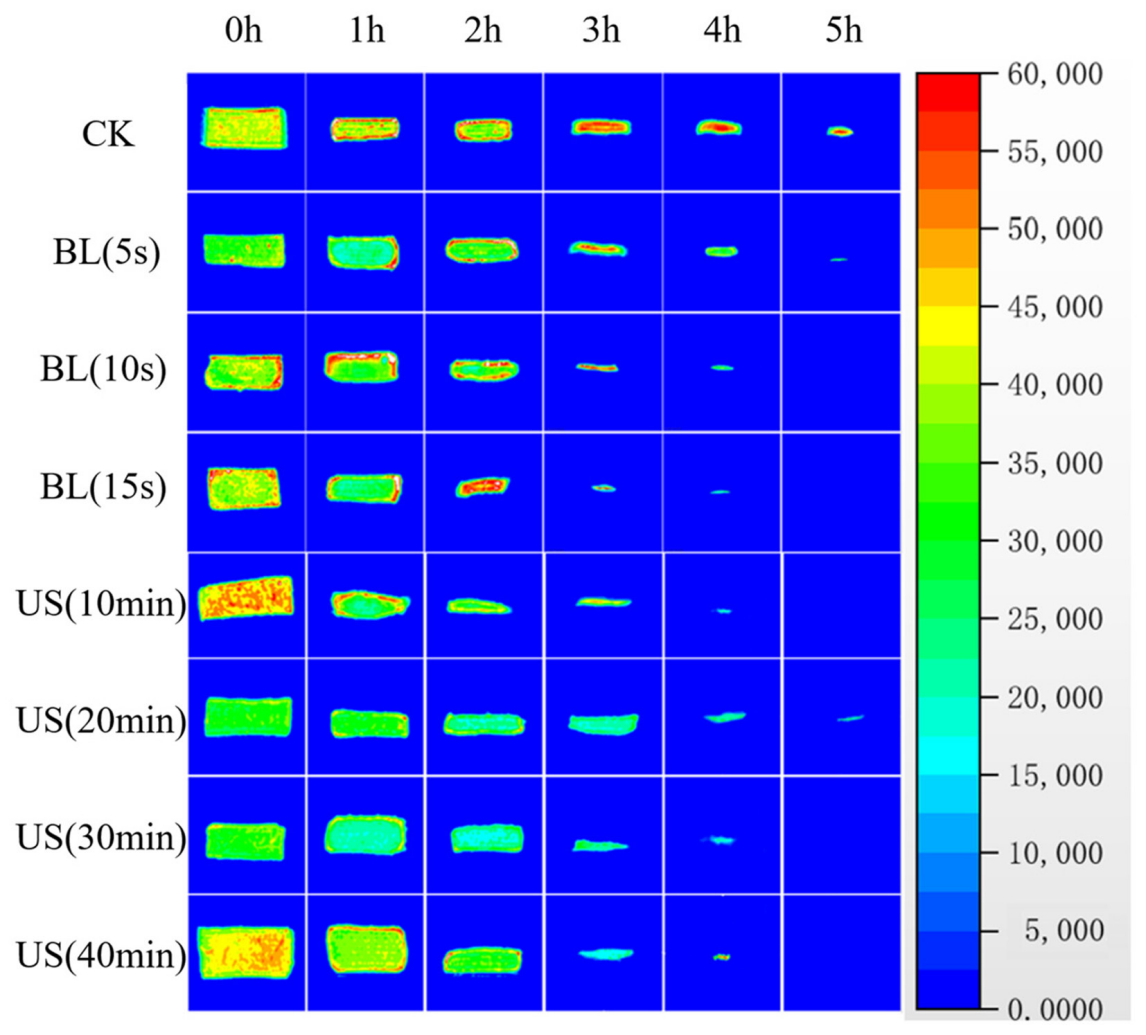
Proton density weighted images of different groups during the drying process.

Figure 5 shows weighted images of the proton density of different groups during the drying process. Vertically, the moisture distribution of different treatment groups at the same drying time is shown, and horizontally, the moisture distribution of the same treatment group at different drying times is shown. Red represents the area with the highest proton density, which indicates the high water content, whereas blue shows the opposite (Zhang et al., 2020). The results show that both BL and US pretreatments can shorten the drying time. In the later stage of CK drying, the internal moisture content is still high. This is caused by the crusting phenomenon, which is caused by the closure of outward diffusion channels caused by surface shrinkage deformation during the moisture diffusion process. This crusting greatly increases the diffusion resistance, prolongs the drying time, and reduces the quality of the material.

From the vertical direction, compared with the CK treatment group, BL and US pretreatment affected the moisture distribution of cantaloupe slices and accelerated their drying rate. Judging from the distribution of the area shown in red, BL mainly acted on the outside, destroyed the cell structure of the outer layer of the cantaloupe tissue, increased the external moisture distribution, reduced the resistance to water diffusion, and shortened the drying time (Ando et al., 2016). In contrast, US pretreatment acted on the inside of the cantaloupe, where cavitation increased the internal water content, formed a higher osmotic pressure that promoted the external migration of water, and shortened the drying time.

### 3.7 Effect of BL and US pretreatments on the texture of cantaloupe slices

The hardness, brittleness, toughness, and other single values measured only by texture cannot completely reflect the crusting phenomenon during the drying process and the influence of pretreatment on uniformity. Therefore, it is necessary to analyze the changes and causes of the observed texture curve in more detail.

**FIGURE 6:**
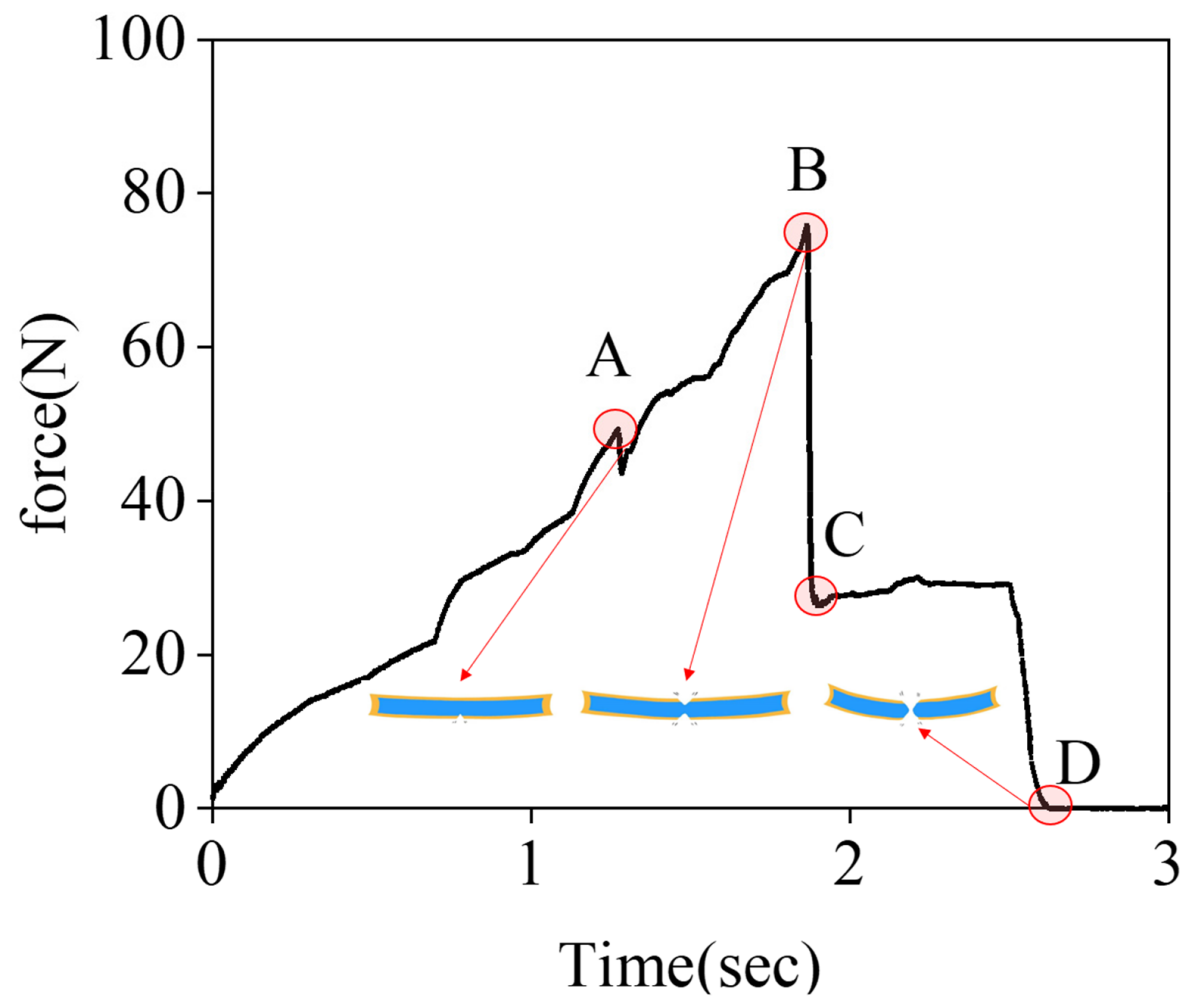
Force evolution curve of cantaloupe during texture.

As shown in Figure 6, two peaks (A, B) were obtained and the region after the highest peak fracture (C–D) generally appeared in the texture curve. During the texture test, the probe applied a force on the cantaloupe which deformed it. At first, the cantaloupe sample will bend downward, the upper surface will shrink, and the bottom surface will stretch. The deformation of the bottom surface exceeds that of the upper surface, reaches the limit position of the extension of the bottom surface, and then, the bottom surface breaks to form the first peak A. Then, as the force continues to be applied, the deformation of the upper surface reached the limit, fracture occurred, and the second peak point B appears. After the upper and bottom surfaces were broken, the pulp part of the cantaloupe sample was still connected, which can be considered a “sandwich layer.” Because its hardness was lower than that of the upper and bottom surfaces, the force required for the deformation of the C–D “sandwich layer” in Figure 6 is lower than at A and B.

The area enclosed by this part is the work required to deform the “sandwich layer” to fracture. The higher the value, the greater the work required to achieve fracture, and the greater the difference between the internal and surface structure of the material, the lower its uniformity.

Table 4 shows hardness A, hardness B, and uniformity S_C‐D_ for each treatment group. Compared with CK, the hardness levels of A and B of the BL and US pretreatment groups were significantly reduced (*p* < .05). BL pretreatment degrades pectin and changes the structure of the material (Oshima et al., 2021). US pretreatment reduces the hardness, softens the texture, and improves the overall quality of the material causing less cell damage (Lagnika et al., 2018). The S_C–D_ of the BL pretreatment group was 0, and that of the US pretreatment group was significantly reduced. This shows that the dried cantaloupe samples in BL and US pretreatment groups had high uniformity, which effectively solved the encrustation phenomenon in CK.

**TABLE 4:** Hardness A, hardness B, and uniformity S_C–D_ of each treatment group.

Sample	Hardness A (*N*)	Hardness B (*N*)	Uniformity (S_C–D_)
CK	30.27 ± 1.47^a^	75.12 ± 2.86^a^	52.41 ± 3.33^a^
BL (5 s)	22.95 ± 2.71^d^	71.25 ± 3.14^ab^	0
BL (10 s)	0	69.18 ± 1.88^b^	0
BL (15 s)	0	60.48 ± 3.52^c^	0
US (10 min)	27.41 ± 2.32^b^	56.71 ± 1.25^c^	0
US (20 min)	24.22 ± 1.64^c^	50.32 ± 3.24^d^	33.98 ± 2.57^b^
US (30 min)	24.29 ± 1.66^c^	40.57 ± 2.9^e^	31.79 ± 2.01^c^
US (40 min)	15.11 ± 2.45^e^	34.93 ± 3.1^e^	0

*Note*: Different values are shown as significantly different (*p* < .05).

## 4 CONCLUSIONS

The effects of BL and US pretreatments on the moisture ratio, drying rate, moisture‐effective diffusivity, color, browning, NMR characteristics, and texture of cantaloupe samples were comprehensively analyzed. Compared with CK, the drying time of BL and US pretreatment groups decreased by 40% and 33.3% at most, respectively. Compared with the drying time of CK, the drying time required to reach equilibrium moisture content in BL pretreated samples (treatment for 5, 10, and 15 s) and US pretreatment samples (treatment for 10, 20, 30, and 40 min) decreased by 6.7%, 24.7%, 40%, 12%, 21%, 26.7%, and 33.3%, respectively. The effective moisture diffusivity of CK was 2.86 × 10^−10^, that of the BL pretreatment group was 2.32 × 10^−9^–2.93 × 10^−9^, and that of the US pretreatment group was 9.96 × 10^−10^–2.22 × 10^−9^. BL and US pretreatments increased the effective diffusion coefficient, reduced the drying time, and improved the drying efficiency. After cantaloupe slices were BL and US pretreated, browning was suppressed to a certain extent during the drying process, which effectively improved the lightness difference (ΔL*) of dried cantaloupe samples and achieved better coloration. LF‐NMR analysis showed that free water was mainly lost during the initial stage of drying, and solidified water was mainly lost during middle and late stages. BL and US pretreatments promoted the diffusion of solidified water and achieved more complete drying. According to MRI analysis, from the perspective of the moisture distribution during the drying process, BL pretreatment mainly acts on the outside, destroys the structure of external cells, and reduces the diffusion resistance; US pretreatment mainly acts on the inside, and the cavitation effect causes the moisture content of each part to increase the formation of osmotic pressure, thus promoting the outward diffusion of water. The texture data define the area enclosed by S_C–D_ as uniform. After BL and US pretreatments, the hardness of dried cantaloupe was lower and the uniformity increased significantly. BL pretreatment for 10 s and US pretreatment for 10 min were identified as optimal treatment protocols, and Us pretreatment was better in comparison. US pretreatment for 10 min could better protect color and inhibit browning while avoiding the crusting phenomenon during the drying process. This study provides a reference for product drying in industrial production. The methods developed can be easily integrated into the production process and better applied in practice.

## AUTHOR CONTRIBUTIONS

**Tiejian Yuan:** Conceptualization (lead); methodology (lead); software (lead); visualization (lead); writing – original draft (lead). **Xiaoyan Zhao:** Supervision (equal). **Chao Zhang:** Visualization (equal). **Peng Xu:** Writing – review and editing (equal). **Xiaoqiong Li:** Writing – review and editing (equal). **Zhentao Zhang:** Funding acquisition (lead). **Junling Yang:** Supervision (equal). **Yaoyang Liu:** Visualization (equal). **Yan He:** Funding acquisition (lead).

## FUNDING INFORMATION

This research was funded by the National Key Research and Developments Program of China (2022YFE0120900), a Special project for the transformation of scientific and technological achievements (No. 2022‐NK‐115), Key research and development projects in Ningxia Hui Autonomous Region (No. 2021BEG03109), and National Natural Science Foundation of China (No. 52176076).

## ACKNOWLEDGMENTS

Authors like to thank to their supervisors, Yan He and Zhentao Zhang, for their guidance during this research.

## CONFLICT OF INTEREST STATEMENT

The authors declare that they have no known competing financial interests or personal relationships that could have appeared to influence the work reported in this paper.

## Data Availability

All data included in this study are available upon request by contact with the corresponding author.
